# Standardised lesion segmentation for imaging biomarker quantitation: a consensus recommendation from ESR and EORTC

**DOI:** 10.1186/s13244-022-01287-4

**Published:** 2022-10-04

**Authors:** Nandita M. deSouza, Aad van der Lugt, Christophe M. Deroose, Angel Alberich-Bayarri, Luc Bidaut, Laure Fournier, Lena Costaridou, Daniela E. Oprea-Lager, Elmar Kotter, Marion Smits, Marius E. Mayerhoefer, Ronald Boellaard, Anna Caroli, Lioe-Fee de Geus-Oei, Wolfgang G. Kunz, Edwin H. Oei, Frederic Lecouvet, Manuela Franca, Christian Loewe, Egesta Lopci, Caroline Caramella, Anders Persson, Xavier Golay, Marc Dewey, James P. B. O’Connor, Pim deGraaf, Sergios Gatidis, Gudrun Zahlmann, European Society of Radiology, European Organisation for Research and Treatment of Cancer

**Affiliations:** 1 Division of Radiotherapy and Imaging, The Institute of Cancer Research and Royal Marsden NHS Foundation Trust, London, UK; 2 Department of Radiology and Nuclear Medicine, Erasmus MC, University Medical Center, Rotterdam, The Netherlands; 3 Nuclear Medicine, University Hospitals Leuven, Leuven, Belgium; 4 Nuclear Medicine and Molecular Imaging, Department of Imaging and Pathology, KU Leuven, Leuven, Belgium; 5 Quantitative Imaging Biomarkers in Medicine (QUIBIM), Valencia, Spain; 6 College of Science, University of Lincoln, Lincoln, Lincoln, LN6 7TS UK; 7 INSERM, Radiology Department, AP-HP, Hopital Europeen Georges Pompidou, Université de Paris, PARCC, 75015 Paris, France; 8 School of Medicine, University of Patras, University Campus, Rio, 26 500 Patras, Greece; 9 Department of Radiology and Nuclear Medicine, Amsterdam, UMC, Vrije Universiteit Amsterdam, Amsterdam, The Netherlands; 10 Department of Radiology, University Medical Center Freiburg, Freiburg, Germany; 11 Department of Biomedical Imaging and Image-Guided Therapy, Medical University of Vienna, Vienna, Austria; 12 Memorial Sloan Kettering Cancer Centre, New York, NY USA; 13 Department of Biomedical Engineering, Istituto di Ricerche Farmacologiche Mario Negri IRCCS, Bergamo, Italy; 14 Department of Radiology, Leiden University Medical Center, Leiden, The Netherlands; 15 Biomedical Photonic Imaging Group, University of Twente, Enschede, The Netherlands; 16 Department of Radiology, University Hospital, LMU Munich, Munich, Germany; 17 Department of Radiology, Institut de Recherche Expérimentale et Clinique (IREC), Cliniques Universitaires Saint Luc, Université Catholique de Louvain (UCLouvain), 10 Avenue Hippocrate, 1200 Brussels, Belgium; 18 Department of Radiology, Centro Hospitalar Universitário do Porto, Instituto de Ciências Biomédicas de Abel Salazar, University of Porto, Porto, Portugal; 19 Division of Cardiovascular and Interventional Radiology, Department for Bioimaging and Image-Guided Therapy, Medical University of Vienna, Vienna, Austria; 20 Nuclear Medicine, IRCCS - Humanitas Research Hospital, via Manzoni 56, Rozzano, MI Italy; 21 Radiology Department, Hôpital Marie Lannelongue, Institut d’Oncologie Thoracique, Université Paris-Saclay, Le Plessis-Robinson, France; 22 Department of Radiology, and Department of Health, Medicine and Caring Sciences, Center for Medical Image Science and Visualization (CMIV), Linköping University, Linköping, Sweden; 23 Queen Square Institute of Neurology, University College London, London, UK; 24 Department of Radiology, Charité Universitätsmedizin Berlin, Berlin, Germany; 25 Department of Radiology, University of Tubingen, Tübingen, Germany; 26 Radiological Society of North America (RSNA), Oak Brook, IL, USA; 27 European Society of Radiology (ESR), Am Gestade 1, Vienna, Austria; 28 European Organisation for Research and Treatment of Cancer, Brussels, Belgium

**Keywords:** Segmentation and standardisation, mDelphi, Region of interest, Organ-specific, Modality-specific

## Abstract

**Background:**

Lesion/tissue segmentation on digital medical images enables biomarker extraction, image-guided therapy delivery, treatment response measurement, and training/validation for developing artificial intelligence algorithms and workflows. To ensure data reproducibility, criteria for standardised segmentation are critical but currently unavailable.

**Methods:**

A modified Delphi process initiated by the European Imaging Biomarker Alliance (EIBALL) of the European Society of Radiology (ESR) and the European Organisation for Research and Treatment of Cancer (EORTC) Imaging Group was undertaken. Three multidisciplinary task forces addressed modality and image acquisition, segmentation methodology itself, and standards and logistics. Devised survey questions were fed via a facilitator to expert participants. The 58 respondents to Round 1 were invited to participate in Rounds 2–4. Subsequent rounds were informed by responses of previous rounds.

**Results/conclusions:**

Items with ≥ 75% consensus are considered a recommendation. These include system performance certification, thresholds for image signal-to-noise, contrast-to-noise and tumour-to-background ratios, spatial resolution, and artefact levels. Direct, iterative, and machine or deep learning reconstruction methods, use of a mixture of CE marked and verified research tools were agreed and use of specified reference standards and validation processes considered essential. Operator training and refreshment were considered mandatory for clinical trials and clinical research. Items with a 60–74% agreement require reporting (site-specific accreditation for clinical research, minimal pixel number within lesion segmented, use of post-reconstruction algorithms, operator training refreshment for clinical practice). Items with ≤ 60% agreement are outside current recommendations for segmentation (frequency of system performance tests, use of only CE-marked tools, board certification of operators, frequency of operator refresher training). Recommendations by anatomical area are also specified.

## Key points

- System certification and specification of image characteristics is recommended for segmentation.
- Both CE marked and verified research tools are allowable for segmentation.
- Operator training and refreshment are mandatory for segmentation in clinical trials and research.
- Pixel numbers within lesion segmented and post-reconstruction algorithms used need reporting.
- Board certification of operators and frequency of re-training need not be mandated.

## Introduction

Quantitative imaging biomarkers (QIBs) provide additional information to visual image interpretation. They may influence decisions regarding disease presence, natural history, optimal treatment planning/delivery, longitudinal measurements through interventions/treatments and during follow-up, recognition of emergence and patterns of resistance to treatment, and of oligo- vs. poly-metastatic disease status. It is therefore essential that QIBs are robust and repeatable. To achieve this, there have been substantial international efforts towards standardising the processes by which images are acquired and analysed [1, 2]. Recommendations for quality assurance and control of image acquisition and analysis protocols are now available so that derived quantitative biomarkers may be compared when implemented across multiple centres and acquisition systems, thus also enabling data pooling in clinical trials [3, 4]. Remarkably, however, a key component of the entire process of imaging biomarker extraction, the segmentation procedure itself (i.e. the partitioning of the data volume into regions-of-interest), has received relatively poor consideration and still mainly relies on human observer’s perception [5, 6], with a large variation in opinion and practice as to where the borders of a lesion or area of interest might lie. Such variability profoundly affects robust and repeatable quantitation of the derived QIBs. For example, inter-site and inter-scanner variations (including from different protocols for the same techniques/tracers/indications, or various machine types even for the same modality) affect the appearance of the image output and thus the resulting segmentation and QIB data.

Repeatable and accurate lesion segmentation is a cornerstone not only for precise biomarker derivation from single image data sets, but also for comparing images acquired at multiple time points (e.g. in dynamic or longitudinal studies) or using different modalities (including various magnetic resonance imaging (MRI) sequences or different positron emission tomography (PET), or single-photon emission computerised tomography (SPECT) tracers). It is also essential where correction for motion during examinations is required and is of particular importance in the delivery of radiation and other focal therapy. Repeatability is potentially improved by automation [7, 8], while semi-automated methods increase accuracy and avoid the variability of manual methods [9]. Automation may employ methods such as adaptive thresholding and region growing, image gradient-based active contours and level sets, unsupervised statistical and clustering classification, supervised machine learning classification and deep learning methods based on convolutional neural networks [10–12]. Robust, repeatable segmentation methods with an audit trail for clinical trials would permit increased automation, thus improving the consistency of biomarker derivation and enabling workflows unaffected by human-borne variability. This would then support the development of standardised and improved algorithms and artificial intelligence (AI) approaches for automated disease assessment.

Image segmentation involves both identification and delineation of a lesion or tissue [13]. Identification is the process of distinguishing the lesion within the image, while delineation involves the definition of its spatial extent [14]. Therefore, factors that significantly affect lesion segmentation are spatial and contrast resolution, image noise, as well as variability in the shape, texture, and perception of pathologies. Although there have been attempts to introduce standards and guidelines, particularly for segmentation in PET imaging [15, 16], there remains a considerable variation in the process of segmentation [17–19] which requires consensus in order to improve the derivation of QIBs in both clinical trials and clinical practice. In MRI, the Standards for Quantitative Magnetic Resonance committee was set up to devise a framework to ensure that quantitative measures derived from MRI data are comparable over time, between subjects, between sites, and between equipment from the same or different vendors. Standardised methodology for segmentation is particularly pertinent with regard to image types (e.g. anatomical vs. functional data sets), 2D versus 3D segmentation, single- vs. multi-lesion analysis, inclusion of lesion penumbra, translation of segmented regions of interest across data sets (e.g. different modalities or time points), criteria to be applied where image registration is not possible, acceptable accuracy for clinical trials, and development of an audit trail for the segmentation process. This work therefore aims to establish a recommended standard for segmentation by consensus reached through a Delphi process between experts. The Delphi method is commonly used where there are variations in definitions [20] or clinical practice [21, 22]. Delphi relies on decisions from a structured group of individuals because they are more accurate than those from unstructured groups [23]. Through the European Imaging Biomarkers Alliance (EIBALL), a subcommittee of the European Society of Radiology (ESR), and the European Organisation for Research and Treatment of Cancer (EORTC), and with endorsement from the North American Quantitative Imaging Biomarker Alliance (QIBA), we therefore sought input from multiple relevant specialities including radiologists, radiographers, nuclear medicine physicians, technologists, medical physicists, computer scientists, and radiation oncologists in order to compile recommendations for standardised segmentation.

## Methods

### Adopting a modified Delphi method

A modified Delphi (mDelphi) process [24] was undertaken by initiating discussion with selected subject content experts. The modification of the Delphi method potentially improves the initial round response rate and provides a solid grounding based on previously known and developed work. In order to include as many primary stakeholders (i.e. experts routinely performing segmentation) as possible in the process, the first survey was circulated widely through the European Society of Radiology and the EORTC imaging groups. The recipients were asked to consider their expertise and interests in segmentation. Participation was considered mandatory for members of the EIBALL subcommittee and the EORTC Imaging Group steering committee.

**Fig. 1:**
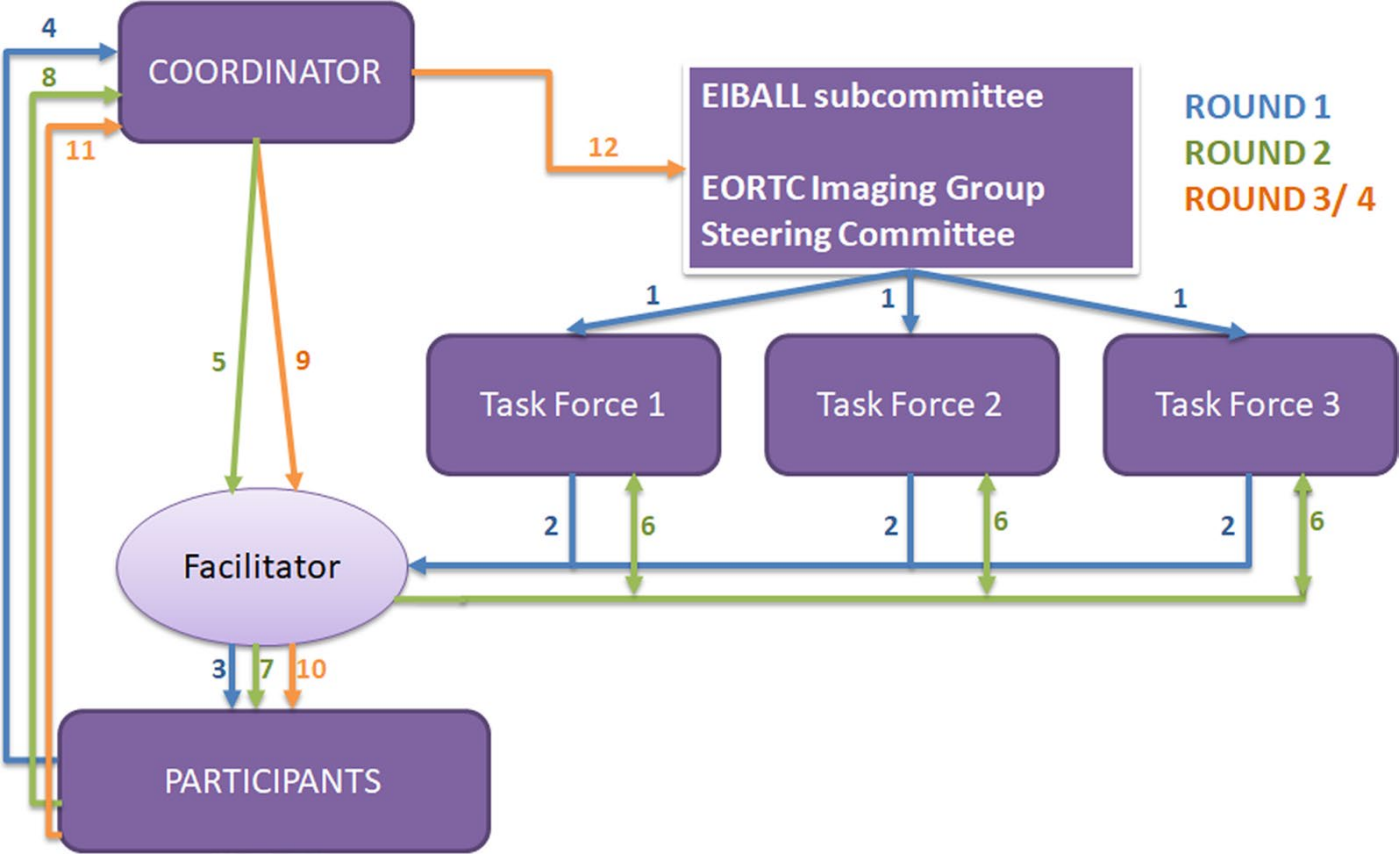
Workflow for mDelphi process: The mDelphi process was initiated at the EIBALL and EORTC Imaging Group steering committee levels. In Round 1 (blue workflow), 3 task forces were assigned (step 1) to deal with (i) modality and image acquisition, (ii) segmentation methodology itself, and (iii) standards and logistical issues. The task forces formulated survey questions and submitted them to the facilitator (step 2) who then distributed them to the participants (step 3), with the results being fed to the co-ordinator (step 4). In Round 2 (green workflow), the facilitator received (step 5) and amalgamated the responses and informed the task forces of the outcome so that they could devise a second round of questions and send them to the facilitator (step 6). These new questions were sent again to the participants of Round 1 (step 7) and responses collected by the coordinator (step 8). For Round 3 and iteratively for Round 4 (orange workflow), the responses received by the facilitator (step 9) were reviewed, and targeted questions were formulated towards achieving consensus and distributed again to participants (step 10). The final responses collected by the co-ordinator (step 11) were circulated to the EIBALL and EORTC Imaging Group steering committees (step 12) for review and analysis

Preliminary discussions at EIBALL and EORTC Imaging Group steering committees identified three overarching areas where consensus was required in order to standardise segmentation: (i) modality and image acquisition, (ii) segmentation methodology itself, and (iii) standards and logistic issues. This led to the creation of three multidisciplinary task forces (one per identified area), each consisting of four persons providing expertise in medical physics, radiology, and nuclear medicine. After internal discussions, each task force submitted questions to a facilitator that covered uncertainties and variations in practice in their respective area. Figure 1 shows the workflow process for the mDelphi process used. Authorship of this manuscript includes all members of the EIBALL and EORTC Imaging Group steering committees who met the criteria for authorship on consensus documents [21].

### Delphi Round 1

This firstly sought to establish the imaging modalities that warranted recommendations for standardised segmentation. Each task force met virtually and developed questions around their assigned topics. The modality and image acquisition taskforce addressed questions for each imaging modality. This related to the respective relevance of instrument performance on image quality for segmentation, image resolution and contrast (including the use of contrast agents, dynamic scans, and radiation dose), post-reconstruction filtering, windowing and thresholding during manual segmentation, and acceptability of artefacts. For hybrid/molecular imaging, additional considerations were addressed related to radiotracer injected activity and time from injection-to-scan acquisition (including dynamic aspects if using dynamic acquisitions and analysis). The segmentation methodology taskforce developed questions around the use of manual, semi-automated and automated segmentation, acceptability of algorithms, how limits and constraints are set. Additionally, organ-specific requirements under categories of brain, head and neck, chest, abdomen, and pelvis and musculoskeletal were interrogated. The standards and logistic task force developed questions around reference standards, evaluation processes and metrics, validation of segmented outlines and operator training.

### Delphi Rounds 2, 3, and 4

After the first round, an anonymised summary of the experts’ forecasts from the previous round was reviewed by the facilitator and circulated to all task forces so that a further round of questions could be devised to refine the results from the previous round (Fig. 1). Rounds 3 and 4 consisted of a repeat of questions from Rounds 2 and 3 where there had been near consensus (60–74% agreed or disagreed). The surveys for Rounds 2, 3, and 4 were circulated to all respondents from Round 1.

### Data analysis

Results from each survey Round were automatically generated by an independent operator also managing the logistics of the survey and were displayed as the percentage of respondents having selected a given answer for every question. These percentages were based on the number of respondents indicating they had sufficient knowledge to answer the question and excluded abstentions. A threshold of 75% was set for consensus agreement as a systematic review had previously shown this to be the median threshold value from 100 Delphi studies [25]. Details of their responses, some of which required further clarification in Rounds 3 and 4, were categorised on a traffic-light system—blue/green, where ≥ 75% of respondents agreed on an item, amber where 60%–74% agreed on an item, and red where < 60% agreed on an item. Therefore, from Delphi Round 1, where 75% of respondents indicated a metric to be important or extremely important, that metric was interrogated further in Round 2. For Delphi Round 2, where 75% of respondents selected one particular answer, this was considered to be a recommendation. Where metrics achieved a 70–74% selection, these were re-interrogated in Round 3 and, for those questions still requiring clarification because of near consensus, subsequently in Round 4 (Fig. 1).

Metrics with a 60–74% selection of a specific answer were considered to currently remain outside a recommended guideline, but as they were identified as items of interest for robust segmentation at the outset, they are regarded as requiring reporting.

## Results

### Response rate and nature of respondents

**Fig. 2:**
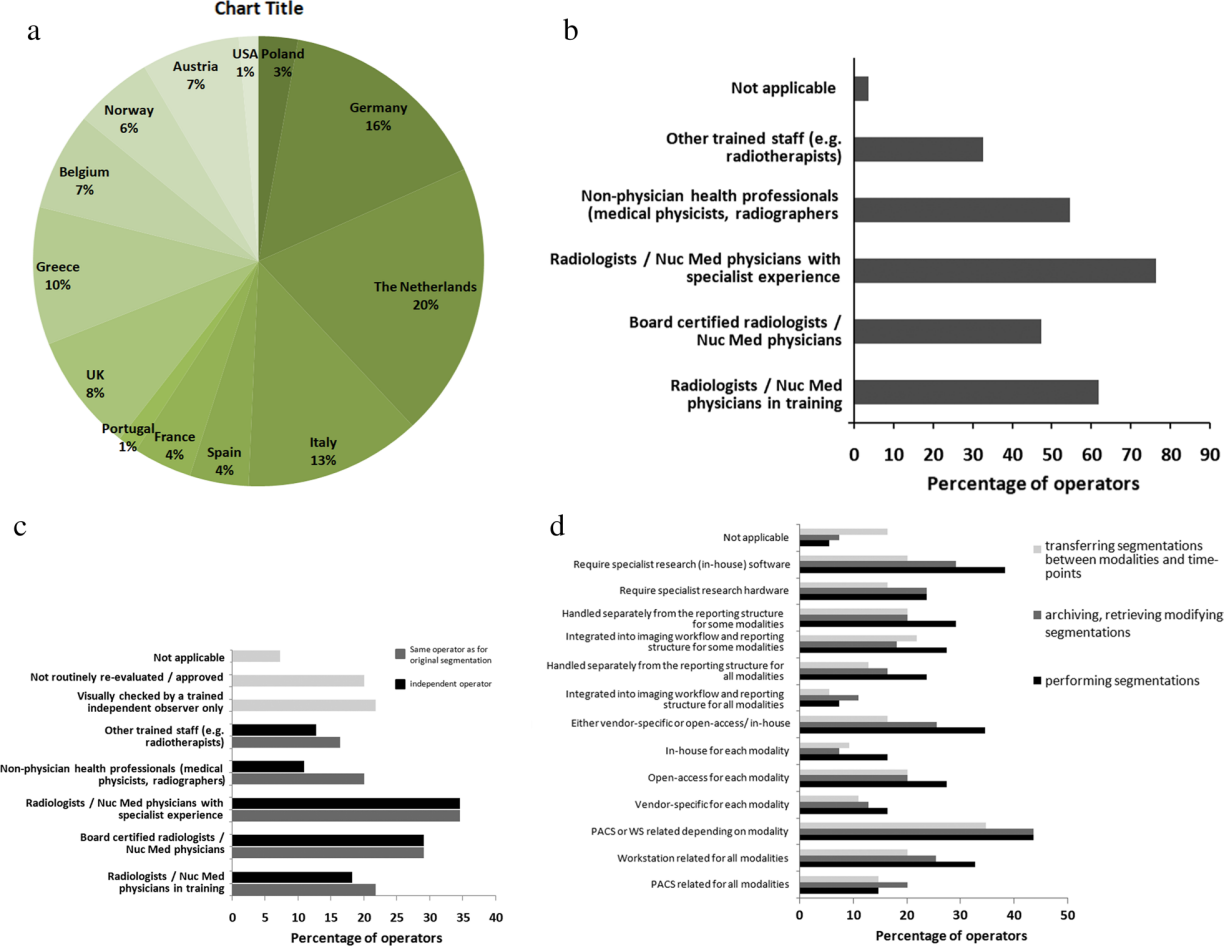
Geographical distribution and experience of respondents to the mDelphi process, and respondents’ use of segmentation: The country of practice of the 58 respondents is given in (**a**). The category of skilled personnel performing segmentation and validating these segmentations in the respondents practice is given in (**b**, **c**), respectively. Tools used for performing the segmentation, archiving, retrieving, and modifying segmentations and for transferring and comparing segmentations between modalities and time points are shown in (**d**)

While seventy-one participants started the survey in Round 1, only 58 responded to the survey questions (40% radiologists, 10% nuclear medicine physicians, 17% medical physicists, 9% radiographer, and 24% outside these specialities, including computer scientists and radiotherapists). The median age range of respondents was 41–50 years and 58% were male. The current experience and practice of the respondents is given in Fig. 2.

### Round 1: establishing factors that require consideration for guideline on standardising segmentation

The factors limiting standardised segmentation were identified as lack of standardised data acquisition (76%), equipment variability within the same modality (68%), lack of standardised post-processing methods (81%), lack of standardised segmentation tools (91%), variation in vendor-specific segmentation software (85%), variability between trained observers (77%), lack of routine performance evaluation (91%), lack of integration into clinical workflow (86%), and lack of ground truth data (71%). The three imaging modalities identified as requiring standardised segmentation were CT and MRI (extremely important or important by 100%), and PET (extremely important or important by 98%). Planar X-ray, ultrasound, and scintigraphy were not identified as needing segmentation standards, and there was insufficient expertise among respondents to draw conclusions for SPECT imaging.

#### Image acquisition

Image contrast was considered extremely important or important by 91%, and background noise by 86%. Adherence to trial protocols was considered extremely important or important by 95%, as well as the availability of standard operating procedures (SOPs) by 97%. Respondents also indicated spatial resolution (88%), contrast resolution (91%), and signal-noise-ratio (SNR) (89%) as important or extremely important regardless of modality and particularly for morphological images. 100% of respondents indicated that the presence/absence of artefacts was critical.

Modality-specific acquisition parameters that were considered extremely important or important for CT were those that affect spatial resolution (94%), radiation dose (81%), and dose of contrast agent (85%). Patient preparation, patient positioning, system performance, scanner type, and radiation dose itself were not considered important. For MRI, imaging sequences (98%) and imaging factors that affect spatial resolution (92%) and contrast dose (80%) also were considered extremely important or important, whereas patient preparation, patient positioning, system performance, and scanner field strength were not. For PET, patient preparation, acquisition timing, administered activity of radiopharmaceutical, scanner camera type, reconstruction method, and system performance were all considered as extremely important or important (89%, 95%, 92%, 85%, 97%, and 89%, respectively), but patient positioning did not matter.

#### Image processing

Image processing parameters considered extremely important or important on CT and MRI were filters applied in reconstruction (87% and 91%, respectively), reconstruction algorithms (92% and 87%, respectively), and embedded post-reconstruction image processing (92% and 87%, respectively). For PET, 89% and 93% considered filters and reconstruction algorithms important. Window and level settings (i.e. display brightness and contrast settings), room lighting, and workstation performance were highlighted by < 75% of respondents for all modalities.

#### Segmentation process

79% of respondents agreed that tools used for segmentation could be a mixture of CE-marked or research tools, that consistency of segmentation must be verified using a reference standard (96%), and that performance must be evaluated by multiple independent observer segmentations (93%). Criteria such as use of solely CE-marked tools, use of a single study to verify consistency of segmentation, and algorithmic evaluation of contours did not reach consensus in Round 1.

#### Reference standards, validation of segmentation and operator training

84% of respondents agreed a reference standard was needed, that comparison between automated and manual outlines was needed where applicable (93%), that segmentation accuracy indices should be used (93%), that automated segmentations results must be verified by human observers (77%), and repeatability tested in individual settings for biomarkers with high inherent variability (93%). The reference standard used when training algorithms should be based on segmentations by multiple trained observers (91%), but board certification or extensive experience for the task was not considered necessary criteria. Validation of manual segmentation was deemed necessary; this could be done by a trained observer performing the segmentation (77%) or an independent trained observer (85%), but, again, these individuals need not be board certified or have extensive experience for the task. Validation of automated segmentation was considered as requiring prior knowledge of anatomy to define constraints (86%), as well as verification by trained observers during algorithm development (95%) and when translated to clinical routine (78%). Operator training was considered necessary by 100% of respondents.

Organ-specific requirements for segmentation, as indicated by respondents with specific expertise in those areas, are given in Table 1.

**Table 1:**
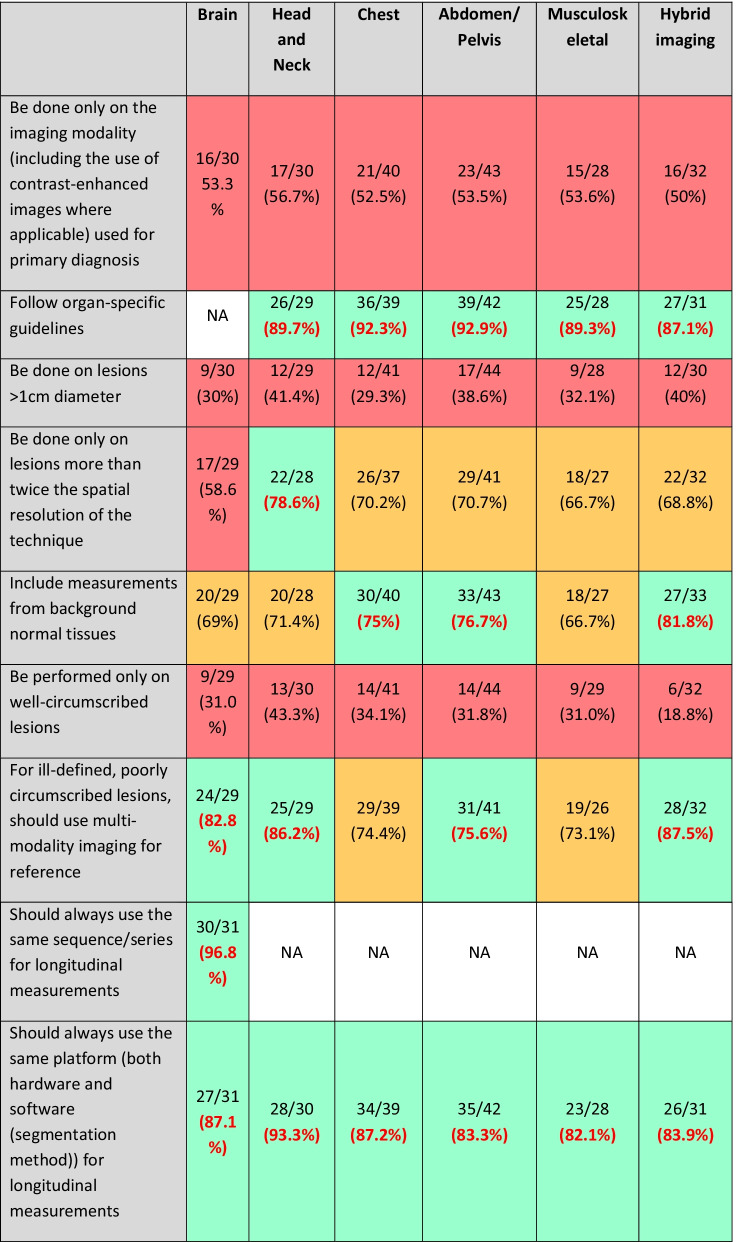
Organ-specific requirements for segmentation from Round 1

Respondents were asked to only answer the sections corresponding to their organ-specific expertise. The items reaching 75% agreement are coded green, those with 60–74% agreements are coded amber and those with < 60% agreement are coded red

### Round 2: establishing consensus opinion for items identified in round 1 for guideline recommendation

Fifty participants responded to Round 2. The numbers of respondents and percentage response for each item, with abstentions, are given in Table 2. Rounds 3 and 4 aimed to reduce the amber category, as it is the objective of a Delphi process to achieve consensus.

**Fig. 3:**
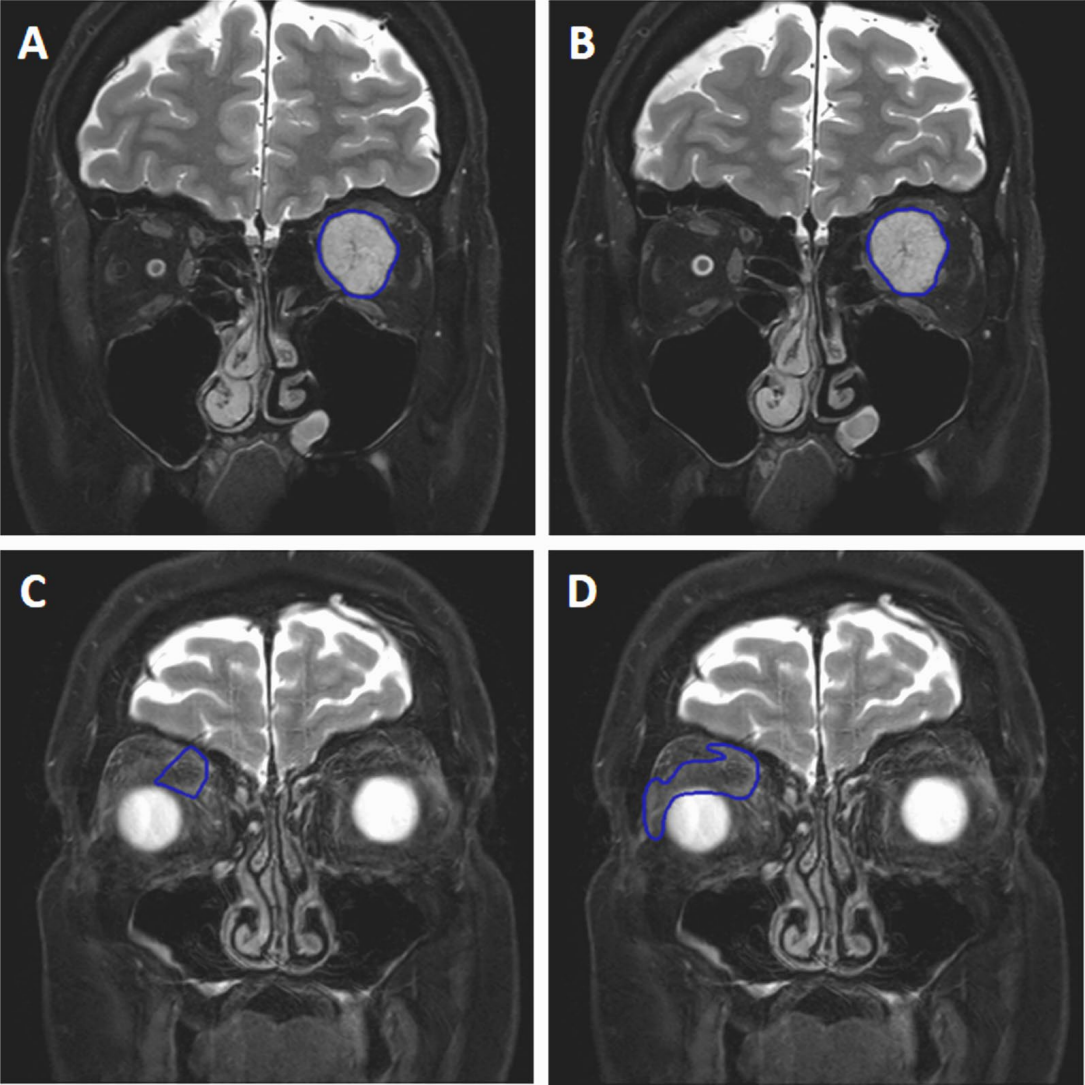
Example of good versus poor reproducibility between observers based on lesion definition: Fat-suppressed coronal T2W images through the orbits in a patient with orbital lymphoma (**A**, **B**) and in a patient with right idiopathic orbital inflammation (**C**, **D**). The reproducibility between the 2 observer segmentations in (**A**, **B**) is excellent (Dice similarity coefficient 0.97), whereas it is poor in the observer segmentations in (**C**, **D**) where the lesion is poorly defined (Dice similarity coefficient 0.5)

- *System performance:* For clinical trials, there was consensus that there must be adherence to guidelines for system performance and that site-specific accreditation was required for all imaging. For clinical research outside trials, this remained ambivalent.
- *Artefacts:* There was consensus that images should be excluded from segmentation if any artefact was propagated across the images being segmented (65%), with a further 11% of respondents indicating that images should be excluded only if more than 5% of the image being segmented was subject to artefacts, so that images without or with < 5% of the image affected by artefact was considered usable by 76% of the respondents.
- *Signal-to-noise ratio (SNR), Contrast-to-noise ratio (CNR), Tumour-to-Background ratio (TBR):* There was consensus that SNR thresholds for assessing a lesion’s suitability for segmentation should be determined by modality, protocol, sequence/tracer, organ or tissue being segmented, with lesion size also being a consideration when setting threshold, and that TBR thresholds set for PET should be study-specific with levels > 2.0 considered as desirable. With regard to CNR, 75% of respondents indicated that it should be equal to or greater than 1.0. The need for specification of characteristics (SNR, CNR, spatial resolution, TBR) of a separate modality being used as a reference when segmenting ill-circumscribed lesions did not achieve consensus. There was also no consensus on specifying the timing of the reference modality in relation to the images being segmented, and this could be at operator’s discretion.
- *Spatial resolution:* When segmenting lesions, there was 95% consensus that the spatial resolution should be documented in pixels/cm, but opinion was divided as to how many pixels were adequate. Only 13% of respondents indicated that < 5 pixels were acceptable.
- *Post-processing:* There was consensus on the following post-processing steps being acceptable: (i) direct reconstruction; (ii) iterative reconstruction; (iii) reconstruction that was machine learning (ML) or deep learning (DL) informed; (iv) window-level (i.e. display brightness and contrast) ranges need to be specified on an individual organ basis (81%); (v) look-up tables are permissible (86%).
- *Reference standards:* There was consensus that that the reference standard should include multiple trained observers, or a combination of trained observer(s) and automated segmentation process.
- *Validation:* 88% of respondents indicated that performance evaluation/validation of a manual segmentation process must be performed by an independent observer(s) or an automated process. However, 76% agreed that an automated process required validation by a human observer(s) and achieve an acceptable similarity score as shown in Fig. 3.
- *Operator training:* Operator training was considered mandatory for clinical trials and clinical research by 100% of respondents. Such training should be either on a study-specific number of data sets or a minimum of 20 data sets. There was a consensus that operator training should be refreshed both for clinical trials (98%) and clinical research (88%). 30% of respondents indicated this should be only for a new trial, 42% recommended an annual refresher, and 26% a 2-yearly refresher. 80% of respondents indicated that a DICE similarity score (as representative of such metrics) of > 0.7 against the reference standard should be achieved, with 68% indicating that this should be > 0.8.

**Table 2:**
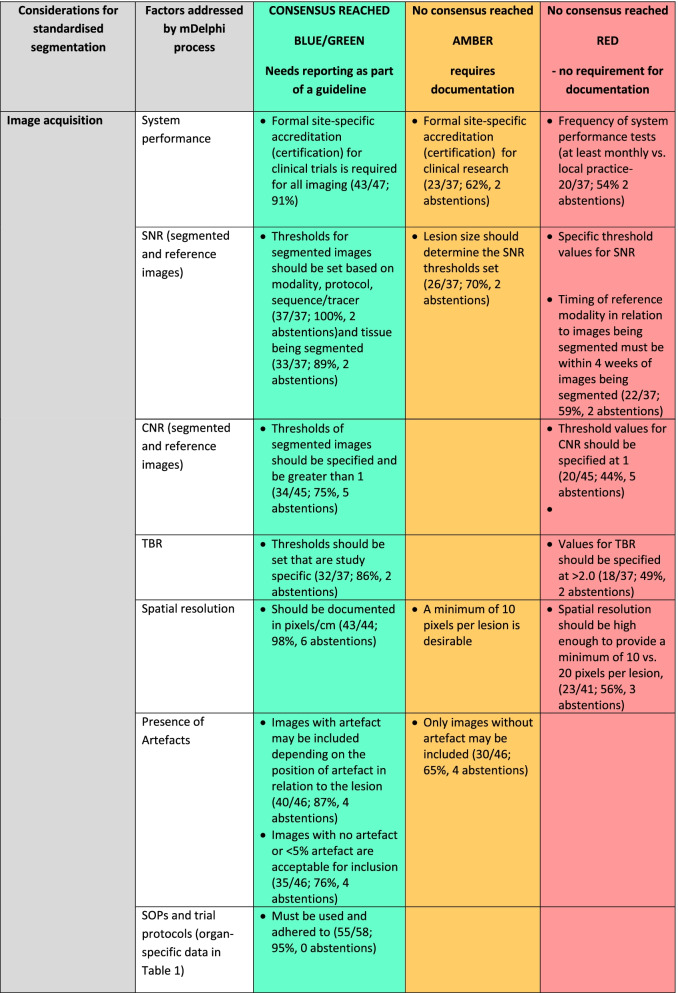

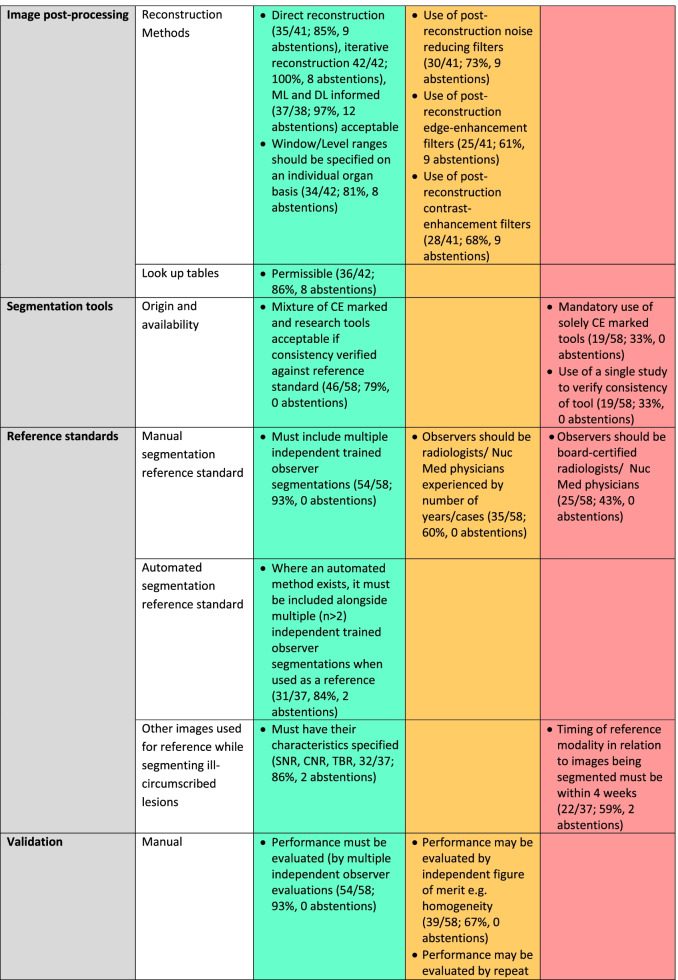

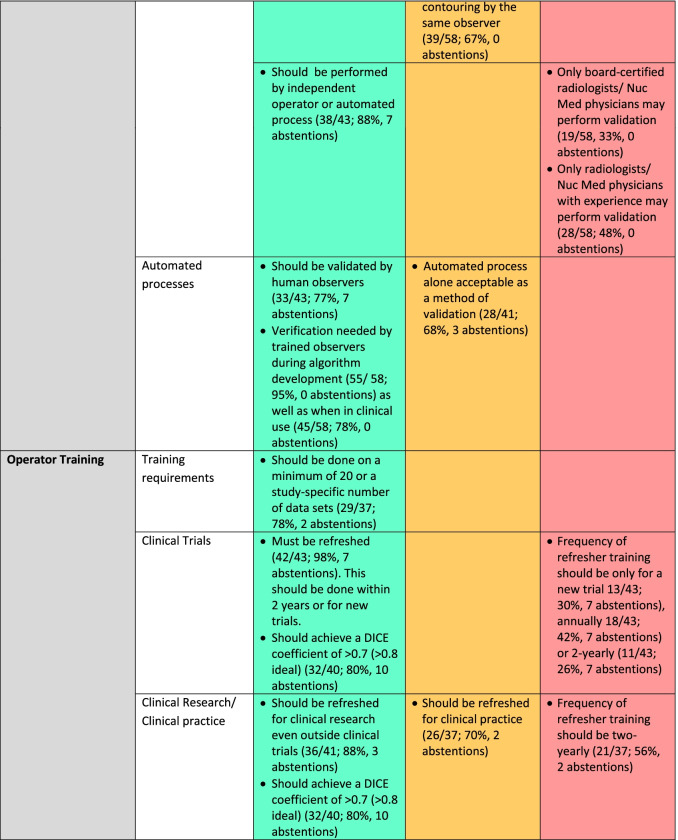
Recommendations for lesion segmentation derived from mDelphi process (Round 1 *n* = 58; Round 2 *n* =  50; Round 3 *n* =  39; Round 4 *n* =  44)

Where > 75% of respondents agree that this should be a standard the factor is classified in the BLUE/GREEN category and needs reporting as part of a guideline.; where 60–75% of respondents identified the factor as important it is listed as AMBER and requires documentation only; where < 60% of respondents identified the factor as important it is designated as a RED category and there is no requirement for documentation

### Rounds 3 and 4: clarifying points from previous rounds where consensus opinion was unclear

**Fig. 4:**
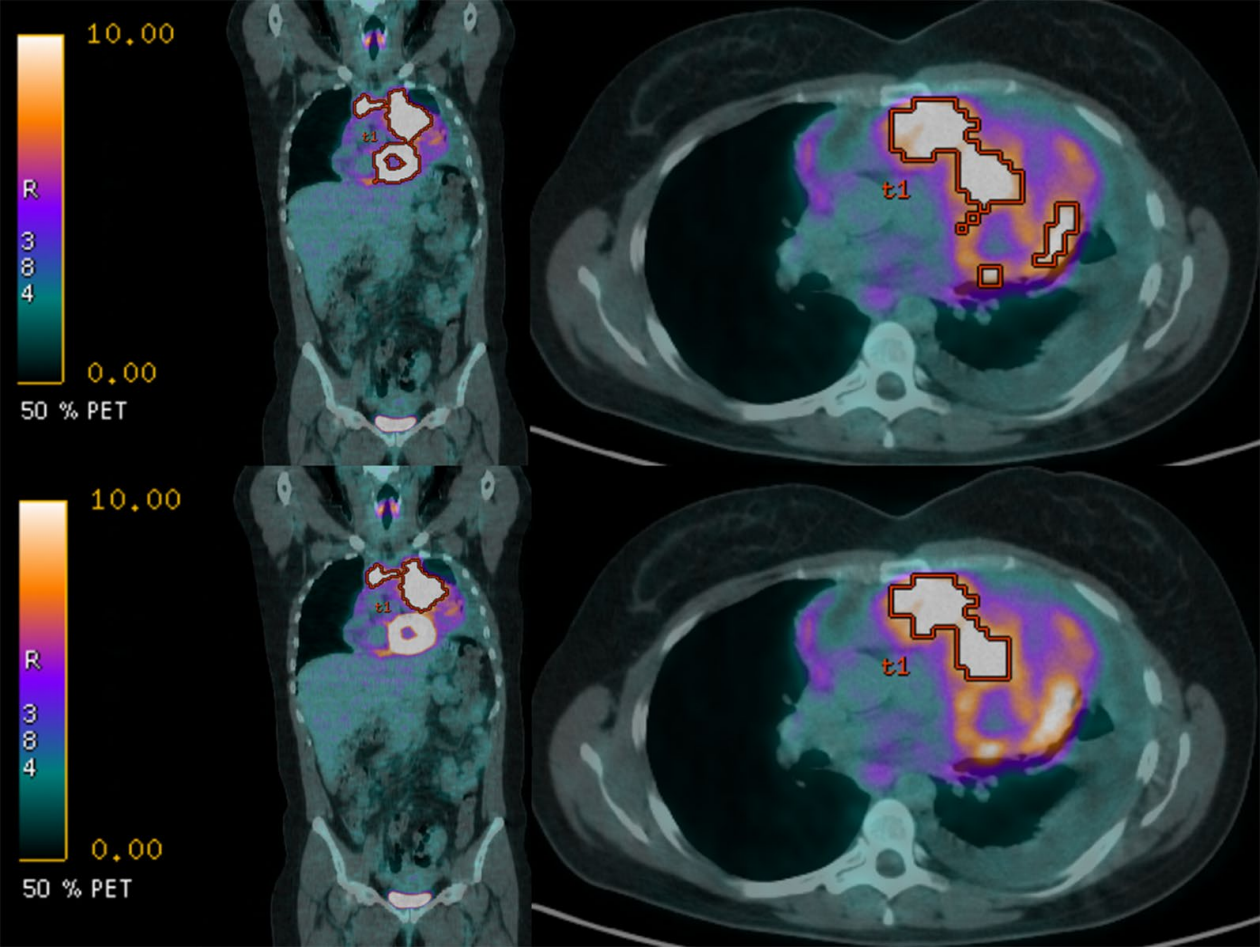
Example of an automated segmentation with manual observer verification and adjustment: Fused coronal (left) and axial (right) [^18^F] FDG PET/CT images of a mediastinal lymphoma following automated segmentation (upper), and following manual adjustment (lower). The automated segmentation used a threshold weight of 0.5 based on an estimated threshold method with the PET VCAR (General Electric) software. It includes the physiological uptake in the myocardium. Manual inspection and adjustment are required to exclude the heart

**Fig. 5:**
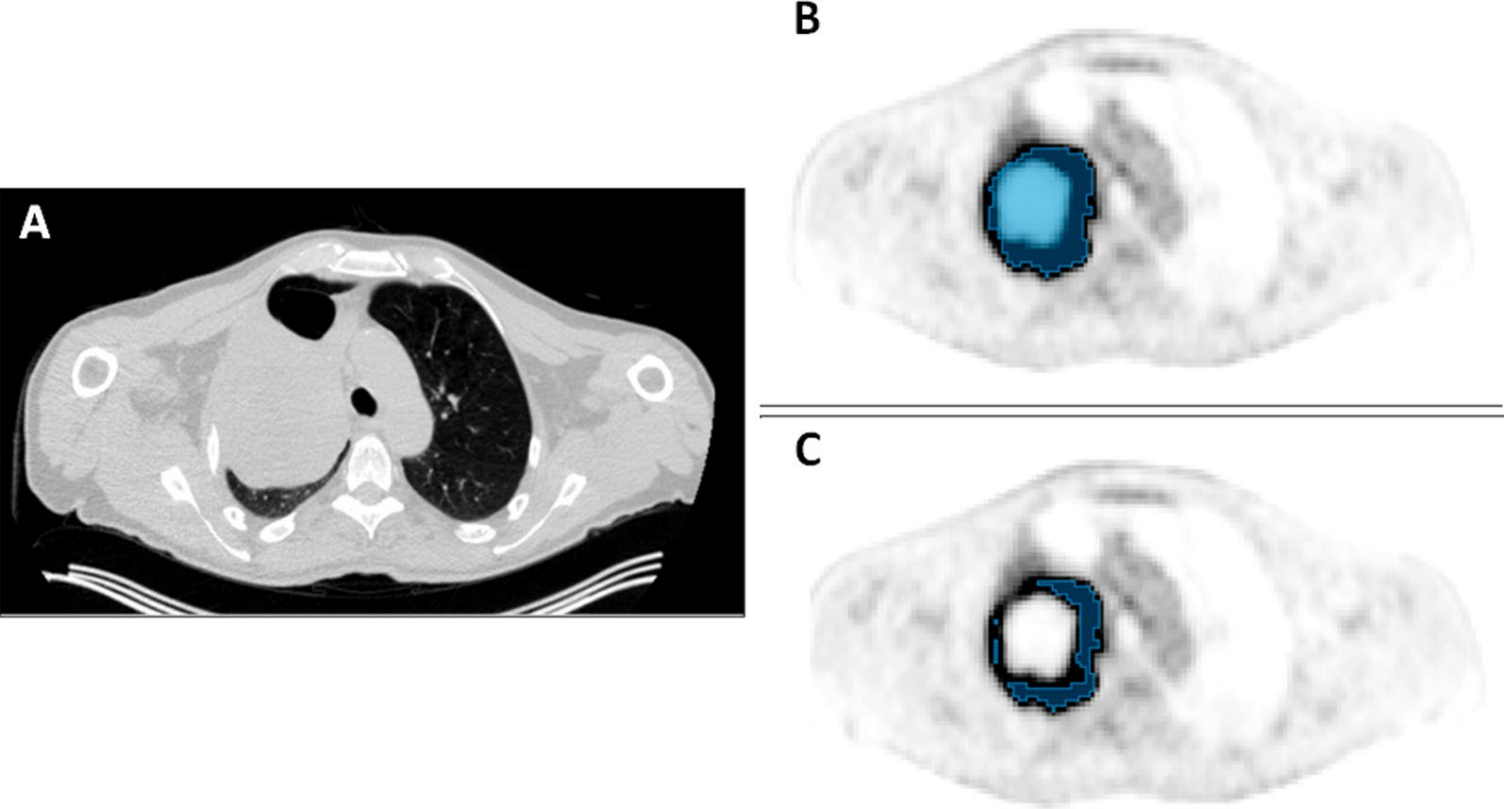
Example of segmentation of selected regions for biomarker derivation requiring specification in standard operating protocols: Axial [^18^F]FDG PET/CT images through the mid thorax in a patient with a T3N1M0 non-small cell lung cancer. On the CT component in (**A**), contrast within the mass is poor. The PET scan (**B**, **C**) differentiates the tumour with a centrally necrotic area. The entire tumour has been segmented in (**B**), but as inclusion of necrosis impacts quantitative biomarker derivation, exclusion of the central necrosis during segmentation, as shown in (**C**), is preferable. The vital tumour volume was delineated using a threshold of 41% of the SUV_peak_ obtained using a sphere of 12 mm diameter, corrected for local background. The gross tumour volume was generated by adding the volumes of the central necrosis to the vital tumour volume

- *System performance:* There was no consensus on the certification needed for clinical research, nor the frequency of local system quality assurance (QA).
- *SNR, CNR, TBR:* There was 100% agreement that SNR threshold should depend on modality, protocol, sequence/tracer and be determined by the organ or tissue being segmented (89%), but lesion size was considered important by only 73% (75% in previous Round). TBR in PET should be set as study-specific (86%), which was considered as more critical than defining a lower limit threshold of 2.0.
- *Spatial resolution:* There was no consensus on whether 10 or more pixels were needed when considering a lesion suitable for segmentation.
- *Image processing:* Factors that fell below the consensus limit for a guideline were the use of filters for smoothing or edge enhancement, noise reduction, and post-reconstruction contrast enhancement (e.g. histogram equalisation).
- *Reference standards:* 84% of respondents agreed that where an automated reference method exists, a reference standard must include such an automated method alongside multiple independent observer segmentations to minimise errors not identified by the automated algorithm (Fig. 4) or to exclude areas within the segmented lesion (such as necrosis) that confound quantitation (Fig. 5).
- *Validation:* There was no consensus on whether an automated process alone could be used for validation of segmentation; only 70% of respondents indicated this as being acceptable.
- *Operator training:* The number of data sets required for operator training should be set by the study, with anywhere between 10 and 40 being needed (91%). 88% of respondents agreed that operator training should be refreshed for clinical research even outside clinical trials, with 70% indicating that this was also needed for clinical practice. There was no consensus on the frequency of refresher training, with the majority (56%) of respondents opting for 2-yearly; 43% suggested annually.

The green highlighted boxes in Table 2 are based on the above data, and they result in guideline recommendations derived from the expert panel’s responses to the mDelphi process.

## Discussion

### The panel

Selection of the experts from the wide international and multidisciplinary EIBALL and EORTC Imaging Group subcommittees avoided methodological weaknesses that can otherwise severely threaten the validity and reliability of Delphi results [26]. Because panel selection critically affects outcome, we sought to include a variety of primary stakeholders, and this is reflected by the fact that only 50% of the respondents were radiologists and nuclear medicine physicians, with the other half being from related specialties, particularly medical physics and computer science which are intimately involved with (automated) segmentation processes. We also ensured that 50% of the panel was represented by members of both the EIBALL subcommittee and the EORTC Imaging Group subcommittee, all of whom are senior members of the imaging community with extensive experience of segmentation in various relevant contexts. Panel selection in a systematic review from 1978 to 2009 of 49 studies employing a Delphi process showed that the median number of panel members was 17 and increased over time. Panels included multiple stakeholders, who were healthcare professionals in 95% of cases [24]. More recently, Delphi studies involving imaging have convened multidisciplinary expert panels of 18–50 relevant stakeholders [27–30].

### System performance

Variability in system performance is well recognised as a factor affecting image biomarker quantitation [31]. Therefore, this must be considered when selecting images for segmentation (a key component of most quantitation approaches), not only in clinical trials and research, but also when making longitudinal measurements in individuals where treatment decisions are based on the results of the measurements. Variability in system performance can affect perception of the boundaries of lesions for both humans and algorithms. For instance, when using automated (e.g. machine learning based) segmentation algorithms where training has been performed on data from quality-controlled devices, data coming from further systems with variable performance are likely to compound segmentation error. The nuclear medicine community have a well-established system for device and site accreditation that ensures that systems meet recognised standards of performance, and that regular quality assurance and control procedures are in place so that these sites and devices are accredited to perform quantitative measurements within clinical trials. Such systems are not routinely in place for CT and MRI, and these require individual site review and approval when participating within trials. Increasingly, however, such procedures are being implemented by triallists in order to pool multicentre data, e.g. CT trials for radiomic analyses [32], MRI trials utilising imaging biomarkers in breast cancer [33, 34], prostate cancer [35] and ovarian cancer [36]. In our survey, there was consensus of a certified requirement for systems performance for clinical trials, though this did not reach consensus for research outside the trial setting.

### Artefacts

Unlike segmentation for radiation therapy planning, where lesion delineation is for the purpose of directing therapy, segmentation for image biomarker quantitation requires detailed attention to the location of artefacts and their likely influence on the data derived from the segmented lesion. Non-ferromagnetic metal implants with attenuation of radiation may, for instance, be particularly problematic in CT. Where artefacts obscure lesion boundaries, thus affecting segmentation, it is inadvisable to extract quantitative information by extrapolating lesion edges. However, although the majority of respondents felt that any level of artefact was unacceptable, accepting no or 5% artefact was felt to be acceptable by 75%. Within clinical trials, this runs the risk of bias at the patient level, where more unwell patients may be excluded because of artefact, or at a lesion level, where the segmented ROI might not encompass the entire lesion and its heterogeneity (for instance if there are marked differences between a more vascular periphery and a more necrotic, cystic central region).

### SNR, CNR, and TBR

There was consensus that SNR thresholds should be set based on modality, organ, and lesion size. Noise correction approaches have been compared under different SNR in terms of reproducibility of diffusion tensor imaging (DTI) and diffusion kurtosis imaging (DKI) metrics [37]. Noise bias correction has a strong impact on quantitation for techniques inherently low in SNR such as diffusion-weighted imaging (DWI), and noise bias can lead to erroneous conclusions when conducting group studies. Noise bias correction significantly reduces noise-related intra- and inter-subject variability and should not be neglected in low SNR studies such as DKI [37].

CNR and TBR profoundly affect lesion edge perception and hence directly impact segmentation and the resulting derived quantitative parameters. For example, in MRI, sequences that provide the highest contrast are generally used for segmentation, and the corresponding ROIs are then copied onto images where the contrast between lesion and background is less striking [38]. In PET, high TBR is similarly advantageous for metabolically active tumours, while posing difficulties in segmentation of lesions with low metabolic activity. Where CNR or TBR is high, automated segmentation may be undertaken with greater confidence as techniques such as thresholding, region growing, and machine learning all then become more robust. Thresholding (fixed, adaptive, or iterative) converts a greyscale image into a binary image by defining all voxels greater than some value to be foreground, and considering all other voxels as background [39]. Partial volume effects (linked to a modality’s spatial resolution vs. the size of a region of interest) critically affect selection of optimal thresholds. In many clinical studies, a value such as a standard uptake value (SUV) of 2.5 (for PET) or an apparent diffusion coefficient (ADC) of 1.0 × 10^–3^ (for DWI-MRI) is set as pre-defined threshold levels to differentiate malignant lesions from benign, but there can be substantial variability across multiple studies even in the same tissue type [40, 41]. The data from this Delphi process indicate that 1.0 is a minimal CNR threshold for radiological images, and 2.0 an acceptable TBR for PET data.

Modifying the acquisition parameters that in clinical practice can be selected by the user can significantly impact CNR. In clinical trials, optimising these parameters to achieve a CNR that enables robust segmentation methodology is thus desirable. Brambilla et al. [42] investigated the effect on CNR when varying acquisition parameters such as emission scan duration (ESD) or activity at the start of acquisition (A(acq)), or object properties such as target dimensions or TBR, which depend uniquely on the intrinsic characteristics of the object being imaged. They showed that the ESD was the most significant predictor of CNR variance, followed by TBR and the cross-sectional area of the given sphere (as test object), with A(acq) found to be the least important. Thus, raising ESD seems to be much more effective than raising A(acq) to increase CNR for improving target segmentation. Moreover, when determining percentage thresholds for segmentation, for targets ≤ 10 mm in diameter, target size was the most important factor in threshold selection followed by the TBR, while for targets ≥ 10 mm, the TBR was more important in threshold selection [42]. This is reflected in our recommendations where selection of targets < 10 mm in diameter is not recommended for extracting quantitative imaging biomarker data.

### Spatial resolution

The spatial resolution of images selected for segmentation is not routinely cited, and our data indicate a need for this although there was no consensus on size thresholds that should be set. The majority of respondents indicated that 5 pixels or less was too few, and that the lower limit should be set somewhere between 10 and 20 pixels within a region of interest in order to capture lesion heterogeneity and be representative enough for biomarker quantitation. Such a lower size limit for the target lesion, which will also depend on the intrinsic resolution characteristics of the modality and instrument is also linked to ensuring that partial volume effects do not significantly affect derived measurements.

### Post-processing

Specifying post-processing methods did not achieve consensus, although it was agreed that organ- and modality-specific window and level methods should be used. Multiple methods appeared acceptable without specification of the use or otherwise of filters for edge enhancement, smoothing, or noise reduction. However, within clinical trials, documentation of these parameters should be enforced as they were deemed important or extremely important in the first Round. These data are not currently recorded, not even in clinical trials.

### Reference standards and validation

As phantoms provide the exact dimensions of the objects in the images, using them is one way to create a surrogate truth for measuring the performance of an algorithm or a complete imaging pipeline. Synthetic images effectively serve as digital phantoms, where the true object size and boundaries are known and can be affected by varying noise, artefacts, or other confounding effects [43]. Alternatively, manually segmented structures can be compared with algorithm-generated segmentations in terms of overlap or boundary differences [44]. This strategy is commonly used, but, because of the variability in human perception, it is important to incorporate as many manual segmentations as possible and combine these segmentations together to form a single statistical ground truth. The widely used Simultaneous Truth and Performance Level Estimation (STAPLE) method estimates the ground truth segmentation by weighing each expert observer’s segmentation depending on an estimated performance level [45]. Our study has emphasised the need for multiple operators for manual segmentation in order to generate a reference standard. The use of multiple operators, or a human operator supplemented by an automated process, was reinforced by our survey, particularly for validation. The input of human operators was deemed essential for validating automated processes during algorithm development and subsequent roll out, together with regular training intervals.

### Operator training

Manual segmentation is highly subjective, and intra- and inter-operator agreement rates (citing years of relevant experience of the individual operators) are often presented in the literature, to indicate both the reliability of the obtained surrogate truths and the level of difficulty of the segmentation problem. Moreover, a manual process is time-consuming and labour-intensive. In one study [46] that involved 18 physicians from 4 different departments, the agreement, defined as a volume overlap of ≥ 70%, was found only in 21.8% of radiation oncologists and 30.4% of haematologic oncologists. Smaller lesions (i.e. < 4 cm^3^) suffer much more from partial volume effects [47], which in a fixed, threshold-based phantom study has been shown to critically depend on lesion size (e.g. vs. imaging resolution), contrast, and noise [48], so that challenges and consistency of operator segmentation are also related to these factors. Our work indicates that operator training is more important than board certification and years of experience, and that refreshing training (e.g. using 20 data sets or more) was important within clinical trials on a per trial basis and was also necessary for clinical research and clinical practice. We also obtained consensus that this performance should be validated against the reference standard and achieve a DICE similarity (as representative of such metrics) score of at least 0.7.

## Conclusion and Recommendations

The mDelphi process conducted under the auspices of the ESR’s EIBALL subcommittee and the EORTC Imaging Group steering committee with endorsement from the Quantitative Imaging Biomarkers Alliance (QIBA) has resulted in a series of consensus statements for standardising image segmentation:

- Formal site-specific accreditation (certification) for clinical trials is required for all imaging.
- Segmentation should follow organ-specific guidelines and standard operating protocols.
- Segmentation should always use the same platform (both hardware and software (segmentation method)) for longitudinal measurements.
- Thresholds for image SNR, CNR, TBR, and spatial resolution on images used for segmentation should be set and specified.
- Only images with < 5% artefact within the image may be considered for inclusion when segmenting.
- Direct reconstruction, iterative reconstruction, and machine and deep learning informed reconstructions are all acceptable when segmenting.
- A mixture of CE-marked and research tools for segmentation is acceptable if their consistency is verified against reference standards.
- Reference standards may be derived through manual or automated methods: a reference standard using manual segmentation must include multiple independent trained observer segmentations; where an automated method exists, it must be included alongside multiple (*n* > 2) independent trained observer results.
- Segmentation of ill-defined, poorly circumscribed lesions should use multi-modality imaging for reference; the other images used for reference while segmenting ill-circumscribed lesions must have their characteristics specified (SNR, CNR, TBR, spatial resolution).
- Segmentations must be validated by human operators or automated processes: for manual processes, this requires multiple independent observer evaluations; for automated processes this requires human observer input during algorithm development and during clinical or trial use.
- Operator training is mandatory for clinical trials and clinical research and requires either a minimum of 20 or a study-specific number of data sets; it should be refreshed every 2 years or for new trials and achieve a DICE coefficient of > 0.7 (or equivalent metric) against the reference standard.

The statements from above constitute guidelines for the standardisation of segmentation in medical imaging, and their adoption should have a positive impact in the development of more reproducible studies and the translation of results into clinical practice for the benefit of patients.

## Data Availability

Not applicable in this mDelphi consensus paper.

## Abbreviations

ADC: Apparent Diffusion Coefficient
CE: Conformite Europeen
CNR: Contrast-to-noise ratio
CT: Computerised Tomography
DKI: Diffusion Kurtosis Imaging
DWI: Diffusion-Weighted Imaging
EIBALL: European Imaging Biomarkers Alliance
EORTC: European Organisation for Research and Treatment of Cancer
ESD: Emission Scan Duration
ESR: European Society of Radiology
FDG: Fluorodeoxyglucose
MRI: Magnetic Resonance Imaging
PET: Positron Emission Tomography
QIBA: Quantitative Imaging Biomarkers Alliance
SNR: Signal-to-noise ratio
SOP: Standard Operating Procedure
SPECT: Single-photon Emission Computerised Tomography
TBR: Tumour-to-Background ratio
